# Rebound-associated vertebral fractures after denosumab discontinuation in a lung cancer patient with bone metastases

**DOI:** 10.1016/j.bonr.2022.101582

**Published:** 2022-05-04

**Authors:** Jolan Dupont, Wesley Appermans, Marian Dejaeger, Isabelle Wauters, Michaël R. Laurent, Evelien Gielen

**Affiliations:** a Geriatrics and Gerontology, Department of Public Health and Primary Care, KU Leuven, Leuven, Belgium; b Department of Geriatric Medicine, UZ Leuven, Leuven, Belgium; c Centre for Metabolic Bone Diseases, UZ Leuven, Leuven, Belgium; d Department of Respiratory Medicine, UZ Leuven, Leuven, Belgium; e Imelda Hospital, Bonheiden, Belgium

**Keywords:** Bone turnover markers, Denosumab, Osteoporosis, Vertebral fractures, Rebound phenomenon

## Abstract

Denosumab is a commonly used antiresorptive treatment in patients with osteoporosis or solid tumours with bone metastases. Upon denosumab discontinuation, a rebound phenomenon can occur that results in an increased (vertebral) fracture risk. This phenomenon is well-known in the setting of osteoporosis but rarely reported in cancer patients with bone metastases discontinuing denosumab. We present the case of a 43-year old women with lung cancer and bone metastases who suffered multiple vertebral fractures after discontinuation of denosumab.

## 1 Introduction

Denosumab is a human monoclonal antibody targeting the receptor activator of nuclear factor kappa-B ligand (RANKL), a key factor involved in osteoclast differentiation, function and survival (Cummings et al., 2009). Next to bisphosphonates, denosumab is a commonly used antiresorptive drug for the treatment of osteoporosis and to prevent skeletal-related events (SRE's) (e.g., pathological fractures) in patients with bone metastases or bone lesions (e.g., multiple myeloma) (Planchard et al., 2018; Terpos et al., 2021; Sanchez-Rodriguez et al., 2020). Denosumab acts by binding RANKL and thereby inhibiting the osteoclast-mediated bone resorption, resulting in a suppression of Bone Turnover Markers (BTM) and an increased Bone Mineral Density (BMD) (Cummings et al., 2009). The required dosage of denosumab depends on the indication for which it is used. When used for the treatment of osteoporosis, a dosage of 60 mg is administered every six months subcutaneously (Cummings et al., 2009). In the context of preventing SRE's, a dosage regimen of 120 mg monthly is suggested (Terpos et al., 2021). Furthermore, clear guidelines on treatment duration of denosumab are lacking in patients with osteoporosis or bone metastases. Nonetheless, it has been demonstrated that denosumab can be given safely for at least ten years (Ferrari et al., 2019). To treat osteoporosis, a “lifelong” treatment duration beyond ten years could be considered, or denosumab can be switched to alternative antiresorptive treatment (Sanchez-Rodriguez et al., 2020). In the context of preventing SRE's, denosumab should be initiated at diagnosis of bone metastases and normally continued indefinitely (Coleman et al., 2020; Planchard et al., 2018). However, in some patients with good prognostic features (such as oligometastatic disease or when achieving complete or good partial responses) denosumab treatment might be interrupted and resumed in case of disease progression (Coleman et al., 2020).

Unlike bisphosphonates – which are incorporated in the bone matrix and remain active for years – the antiresorptive effect of denosumab is readily reversible (Cummings et al., 2009). Data from osteoporosis trials demonstrated that discontinuation of denosumab leads to a rapid increase of BTMs in the first several months after wash-out period (six months) and this coincides with a rapid decrease in BMD (Tsourdi et al., 2020; Cummings et al., 2018). Levels of BTMs or BMD reach pre-treatment levels or worse within 24 months after denosumab discontinuation (Tsourdi et al., 2020; Cummings et al., 2018). Accordingly, analysis of the FREEDOM Extension trial suggests an elevated risk of vertebral fractures after discontinuation of denosumab (the so-called rebound-associated vertebral fractures of RAVFs) compared to on-treatment (Cummings et al., 2018). There was an increased risk for multiple vertebral fractures, which is the highest in persons with pre-existing vertebral fractures sustained before or during treatment with denosumab (Cummings et al., 2018). Besides vertebral fractures, recent data also reported hip fractures after denosumab discontinuation (Tsourdi et al., 2020). Experiences in patients with osteoporosis demonstrated that these RAVFs occur already after a short off-treatment period, about 8–16 months after final administration (Tsourdi et al., 2020; Anastasilakis et al., 2017; Florez et al., 2019; Burckhardt et al., 2021). Despite being well-known in osteoporosis, RAVFs are rarely reported in the context of bone metastases (Tyan et al., 2019). In the present paper, we describe a case of RAVFs in a patient with a history of lung cancer and bone metastases who developed multiple vertebral fractures following the discontinuation of denosumab.

## 2 Case

A 43-year old woman presented to the outpatient metabolic bone diseases clinic in February 2021 with severe lumbar back pain that started three months prior to the visit. The patient had a history of pulmonary adenocarcinoma with Anaplastic Lymphoma Kinase translocation and bone metastases (vertebra L3, peduncle Th12, ribs, scapula and pelvis), diagnosed in October 2013. She was successfully treated with ceritinib, leading to a still ongoing deep remission since 2015. According to European Society For Medical Oncology (ESMO) guidelines, bone-targeted therapy should be started upon diagnosis of bone metastases (Coleman et al., 2020). However, in the present case dental work was required prior to therapy initiation and start of denosumab was delayed.

**Fig. 1:**
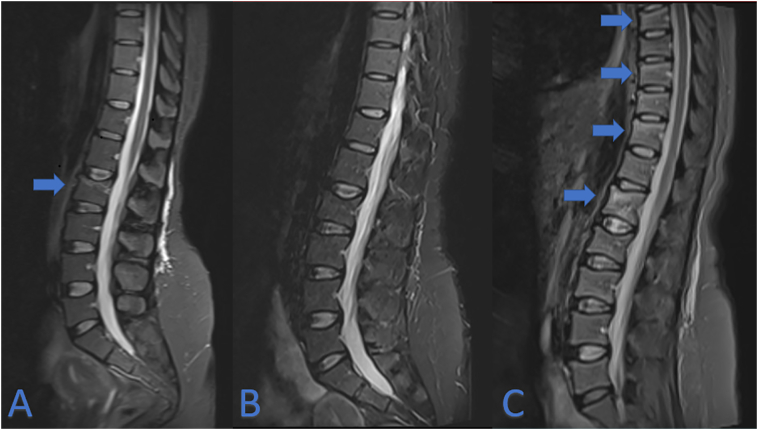
MRI T2-images of the lumbar spine region (A) April 2014, (B) May 2019, (C) February 2021. Blue arrows indicate bone oedema compatible with recent fractures. In A: L2. In C: L1, Th7, Th9 and Th11.

**Fig. 2:**
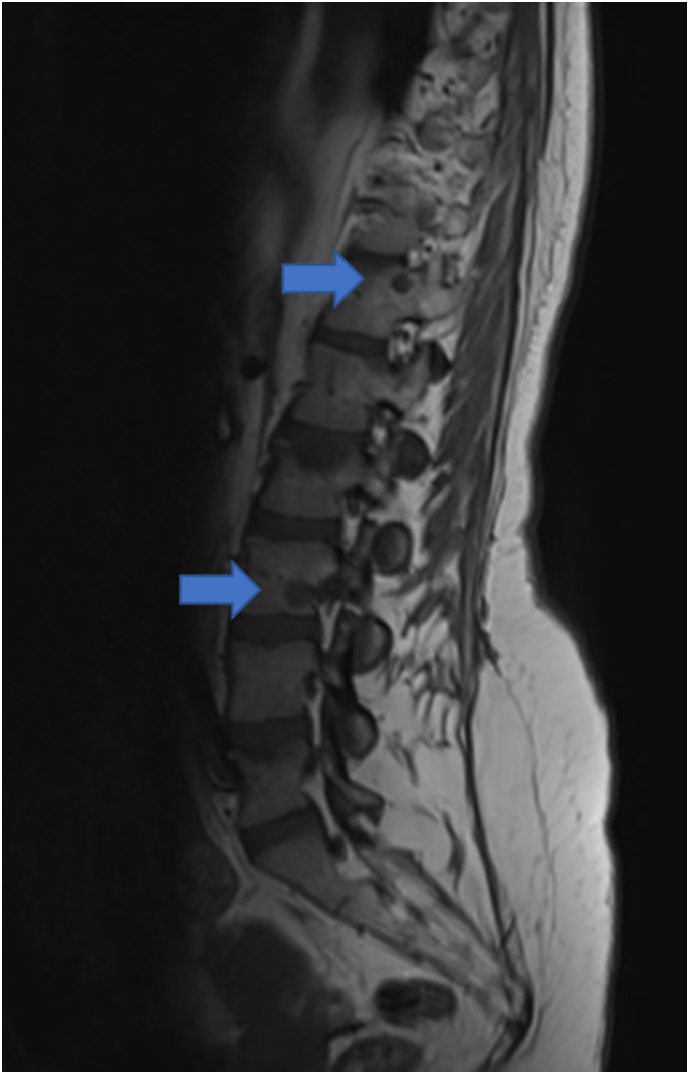
MRI T1-image of the lumbar spine April 2014. Blue arrows indicate T1 hypo-intense metastatic lesions at peduncle of Th12 and corpus of vertebra L3.

Unfortunately, in April 2014, the patient suffered from back pain and a Magnetic Resonance Imagining (MRI) of the spine showed a semi-recent vertebral fracture of L2 with bone marrow oedema and a height loss of 35%, Genant's criteria grade 2 (Fig. 1, Panel A). Moreover, older fractures without oedema were present in vertebrae T12 and L5 (height loss of 10%, Genant's criteria grade 0). All these fractures appeared to be osteoporotic. In contrast, the vertebral metastatic bone lesions were observed in the peduncle of Th12 and the corpus of vertebra L3, as demonstrated by the hypo-intense signal on T1 images (Fig. 2). Subsequently, monthly subcutaneous administration of denosumab 120 mg was started in May 2014 in order to prevented new skeletal events related to the bone metastases. It was interrupted from June to October 2016 due to hypophosphatemia (0.14 mmol/L [reference: 0.81–1.54 mmol/L]) and hypocalcaemia (1.93 mmol/L [2.15–2.55 mmol/L]). At that time, she had a normal kidney function with estimated Glomerular filtration Rate (eGFR CKD-EPI) of 120 mL/min/1.73m^2^ and normal 25-OH vitamin D levels 45.5 μg/L (11.0–60.0 μg/L) with increased levels of parathyroid hormone as high as 204.2 ng/L (14.9–56.9 ng/L).

Upon correction through supplementation, therapy with denosumab was resumed. After six years, denosumab was discontinued due to a longstanding deep remission, compatible with the 2020 ESMO clinical guideline (Coleman et al., 2020). Last administration took place in April 2020. In December 2020, the patient developed atraumatic acute lower back pain. In February 2021, new fractures of vertebrae Th7, Th9, Th11 and L1 were diagnosed on MRI (Fig. 1, panel C), which weren't present on a previous MRI of the spine in 2019 (Fig. 1, panel B). These new fractures were characterised by bone marrow oedema on T2-weighted images, thus suggestive for a recent onset. Accordingly, BTMs were significantly elevated with C-telopeptide of type I collagen (beta-CTx) as high as 1826 ng/L (reference: ≤573 ng/L premenopausal) and Procollagen type 1 N-terminal propeptide (P1NP) up to 304.0 μg/L (reference: 18.0–83.0 μg/L premenopausal). Dual-energy X-ray absorptiometry showed a lumbar spine T-score of −1.6 and a femoral neck T-score of −1.3. A new oncologic work-up with thoracic-abdominal computer tomography (CT) showed stable remission without any sign of relapse or progressive bone metastases. Therefore, these findings are suggestive for RAVFs after denosumab discontinuation. In the presented case, prompt treatment with a dose of 5 mg zoledronate was started. Nonetheless, the patient suffered an additional asymptomatic fracture of Th5, diagnosed on CT-scan three months following the zoledronate administration. At the follow-up in April 2022 – approximately one year after administration of zoledronate – no additional fractures were found but BTM's were still elevated with levels of beta-CTx at 524 ng/L and P1NP at 42.0 μg/L. Accordingly, a second administration of zoledronate was planned.

## 3 Discussion

This report described the case of a 43-year old woman with lung cancer and bone metastases (vertebra L3, peduncle Th12, ribs, scapula and pelvis) suffering from four recent spontaneous RAVFs, developed only eight months after discontinuation of denosumab. These rebound-fractures of vertebrae Th7, Th9, Th11 and L1 did not show any sign of metastatic bone disease progression. RAVFs are a well-known phenomenon in osteoporosis with an estimated incidence of 8–10% after denosumab discontinuation, but are rarely described in cancer patients with bone metastases discontinuing denosumab (Tsourdi et al., 2020; Anastasilakis et al., 2021a). To the best of our knowledge, we are aware of only one other case report of Tyan et al., reporting a patient with bone metastases of lung cancer, who suffered seven RAVFs around 15 months after discontinuation for dental work (Tyan et al., 2019). Similar to our case, the fractures were multiple, showed no signs of oncologic disease progression and bone densitometry at the moment of diagnosing RAVFs showed T-scores in the normal to osteopenia (<−1.0 & >−2.5) range. Also, timing of RAVFs in our case and the Tylan et al. case is within the range of 8–16 months after final administration of denosumab, as previously described in osteoporosis literature (Tsourdi et al., 2020; Anastasilakis et al., 2021a). In contrast to our case, the patient of Tyan et al. did not have pre-existing vertebral fractures, a risk factor to develop multiple RAVFs after denosumab discontinuation (Cummings et al., 2018; Tyan et al., 2019). Upon review of MRI images of our patient – prior to treatment with denosumab in 2014 – two old osteoporotic vertebral fractures were already present prior to therapy with denosumab. This suggests that the patient described in the present case report suffered from osteoporosis prior to her cancer diagnosis.

What might determine that RAVFs upon denosumab discontinuation are rarely reported in cancer patients, compared to osteoporosis patients? It could be that they occur less often.

On the one hand, the recommended *dosage* of denosumab to prevent SRE's is remarkably higher compared to the dosage for osteoporosis treatment (120 mg monthly vs. 60 mg every six months). However, for the treatment of osteoporosis, the dosage of six-monthly 60 mg is deemed sufficient to maximally improve BMD, with no additional gains on BMD to be expected above this dosage (Mandema et al., 2014). Nonetheless, the effect on suppressing BTM's might be different. In a study of Lipton et al. denosumab was administered to cancer patients with bone metastases at a dosage of 120 mg every four weeks and found to suppress urinary-N-telopeptide/creatinine ratio (a specific BTM) adequately after a median time of 13 days (95% confidence interval (CI), 10 to 29 days) (Lipton et al., 2007). The same authors simulated that 120 mg every 4 weeks would result in 95% of patients achieving over 90% suppression of uNTx/Cr, whereas lower dosages (e.g., 30 mg every 4 weeks, which is still higher than the recommended dosage for osteoporosis treatment) would only manage to reach a similar suppression of BTM's in approximately 87% of patients (Lipton et al., 2007). It is unclear whether a deeper suppression of BTM's on therapy contribute to more/less rebound increase of BTMs after denosumab discontinuation and how this affects the risk of RAVFs. Moreover, *pharmacokinetics* might be different in both dosages. Denosumab has a half-life of approximately 26 days (Anastasilakis et al., 2021a). Gibiansky et al. determined that dosage of 120 mg every 4 weeks resulted in a steady state of cancer patients with bone metastases within 4–5 months and serum levels declining over 4–5 months after the final dosage (Gibiansky et al., 2012). In comparison, higher maximal serum concentrations of denosumab were reached with the oncologic dosage when compared to the pharmacokinetics of 60 mg every six months in osteoporosis patients (Sutjandra et al., 2011). It is not excluded that a higher maximal serum concentration might interfere with the risk of developing RAVFs after treatment.

On the other hand, *prevalence of denosumab discontinuation* can be different in both settings. There are no data on exact incidence of denosumab discontinuation, but – as briefly mentioned in the introduction – optimal treatment duration of denosumab is often debated. In the setting of osteoporosis, denosumab usage was often re-evaluated every five years and in case of low fracture risk discontinuation was considered (Tsourdi et al., 2017). However, with the increasing knowledge of rebound-phenomena and RAVFs but a lack of data to support an optimal treatment regimen, a pragmatic approach of “lifelong” treatment or an alternative treatment considered upon discontinuation might be at place (Tsourdi et al., 2020). In comparison, in cancer patients with bone metastases treated with denosumab, discontinuation of denosumab is only considered in case of successful anti-cancer treatment with at least 24 months of remission (Coleman et al., 2020). Moreover, in line of the previous remark, the *life expectancy* between both types of patients might be different. Cancer patients with bone metastases might not live long enough to allow consideration of denosumab discontinuation whereas osteoporosis patients might have a longer lifespan ahead. Therefore, it is hard to tell if RAVFs are indeed more or less prevalent in cancer patients.

How should we treat or prevent RAVFs? A thorough risk-benefit analysis should be done before heading over to stopping denosumab. In case the decision is made to discontinue denosumab, data in osteoporosis patients suggest that after discontinuation, the elevation of BTMs and lowering of BMD should be prevented in order to avoid RAVFs (Tsourdi et al., 2020). This might be done by the administration of bisphosphonates to counteract the rebound-phenomenon (Sølling et al., 2020). The European Calcified Tissue Society (ECTS) position paper recommends giving an alternative antiresorptive treatment like alendronate, when treated with denosumab for a short period of time (<2.5 years) or zoledronate when denosumab is administered for a longer time (Tsourdi et al., 2020). Risedronate has not proven to be effective in preventing BMD loss (Laroche et al., 2020; Davidoff and Girgis, 2020). Optimal timing is suggested to be 6 months after the final denosumab administration, combined with monitoring of BTMs and prompt retreatment with bisphosphonates in case the BTMs exceed the mean value in healthy premenopausal women (Tsourdi et al., 2020). In case RAVFs have already occurred, ECTS is in favour of initiating alternative antiresorptive treatment (e.g., bisphosphonates), prompt re-initiation of denosumab or a combination of denosumab and teriparatide (Tsourdi et al., 2020). Monotherapy of teriparatide is not recommended by the ECTS but might also be useful according to a recent paper on this topic (Anastasilakis et al., 2021b). In the case presented in this manuscript, zoledronate was given but could not fully prevent occurrence of an additional RAVF. This is in line with similar cases in the osteoporosis setting, with one report describing a patient who suffered RAVFs and restarted treatment with denosumab only to suffer further RAVFs several months later (Niimi et al., 2020). Even when denosumab is promptly re-initiated or alternative treatment with osteoanabolic romosuzumab is administered before the occurrence of RAVFs but after denosumab discontinuation, RAVFs may still appear (Anastasilakis et al., 2021c; Kashii et al., 2020). Indeed, randomized clinical trial data comparing zoledronate administration 6 months vs 9 months after denosumab discontinuation demonstrated that, irrespective of the timing, zoledronic acid did not fully prevent loss of BMD in patients discontinuing denosumab but still remains superior to a scenario without alternative treatment (Sølling et al., 2020). Along the same line, in our case the BTMs were still elevated at the follow-up less than one year after administration of zoledronate. As such, more frequent antiresorptive treatment might be required to counteract the rebound phenomenon.

In conclusion, this case emphasizes that RAVFs not only exist in patients with osteoporosis but also in cancer patients with bone metastases discontinuing denosumab. The case calls to action for clinicians to well-consider risk and benefits upon discontinuing denosumab in cancer patients with bone metastasis. Nonetheless that in the presented case an underlying pre-existing osteoporosis is not to be excluded, the importance of preventing and detecting RAVFs in cancer patients is crucial, since prompt alternative antiresorptive treatment might be required. Future research regarding RAVFs should also include cancer patients with bone metastases, in order to provide supporting data to guide clinicians on the decision whether or not to discontinue denosumab in these patients.

## Ethics approval

Informed consent was approved by the ethics committee of UZ/KU Leuven (s65243).

## Consent for publication

Written informed consent was obtained from the patient described in this manuscript.

## Declaration of competing interest

JD has received a research grant (11A9320N) and travel support from 10.13039/501100003130Research Foundation Flanders (FWO) as well as a scholarship from Eli Lilly. ML has received conference support and lecture fees from Amgen (related to denosumab), lecture fees from Menarini, and consultancy fees from Alexion, Kyowa Kirin, Sandoz, Takeda and UCB. EG has received lecture fees from Amgen and Takeda, consultancy fees from Alexion and UCB and travel support from 10.13039/100002429Amgen and 10.13039/100011110UCB. WA, MD and IW have no conflicts of interest to declare.
